# Quantitative Mechanistic Modeling in Support of Pharmacological Therapeutics Development in Immuno-Oncology

**DOI:** 10.3389/fimmu.2019.00924

**Published:** 2019-04-30

**Authors:** Kirill Peskov, Ivan Azarov, Lulu Chu, Veronika Voronova, Yuri Kosinsky, Gabriel Helmlinger

**Affiliations:** ^1^M&S Decisions, Moscow, Russia; ^2^Computational Oncology Group, I.M. Sechenov First Moscow State Medical University of the Russian Ministry of Health, Moscow, Russia; ^3^Quantitative Clinical Pharmacology, Early Clinical Development, IMED Biotech Unit, AstraZeneca Pharmaceuticals, Boston, MA, United States

**Keywords:** immuno-oncology, mechanistic models, tumor vs. immune system, systems pharmacology, pharmacokinetics, pharmacodynamics, molecular and cellular biomarkers

## Abstract

Following the approval, in recent years, of the first immune checkpoint inhibitor, there has been an explosion in the development of immuno-modulating pharmacological modalities for the treatment of various cancers. From the discovery phase to late-stage clinical testing and regulatory approval, challenges in the development of immuno-oncology (IO) drugs are multi-fold and complex. In the preclinical setting, the multiplicity of potential drug targets around immune checkpoints, the growing list of immuno-modulatory molecular and cellular forces in the tumor microenvironment—with additional opportunities for IO drug targets, the emergence of exploratory biomarkers, and the unleashed potential of modality combinations all have necessitated the development of quantitative, mechanistically-oriented systems models which incorporate key biology and patho-physiology aspects of immuno-oncology and the pharmacokinetics of IO-modulating agents. In the clinical setting, the qualification of surrogate biomarkers predictive of IO treatment efficacy or outcome, and the corresponding optimization of IO trial design have become major challenges. This mini-review focuses on the evolution and state-of-the-art of quantitative systems models describing the tumor vs. immune system interplay, and their merging with quantitative pharmacology models of IO-modulating agents, as companion tools to support the addressing of these challenges.

## Introduction

Immunotherapy of cancer has had a long history of development, starting from pioneering efforts in using coley toxins to treat patients—a therapeutic approach named after Dr. William Coley (1). Even though these earlier efforts never turned into a standard treatment, further investigations on the relationships between tumor cells and the immune system led to discoveries which unveiled fundamental principles underlying cancer progression, such as immune surveillance (2, 3), cancer dormancy (4), cancer immuno-editing (5), and the cancer immunity cycle (6). These discoveries were foundational for clinical successes and corresponding regulatory approvals in recent years, of therapies targeting the CTLA-4, PD-1, and PD-L1 immune checkpoints. In the wake of these successes, there has been an explosion in the development of immuno-modulating, anti-cancer pharmacological modalities, leading to the initiation of, literally, thousands of clinical trials (7, 8). However, from the discovery phase to late-stage clinical testing and regulatory approval, challenges in the development of immuno-oncology (IO) drugs are multi-fold and complex (9), with related complexities in the design of clinical trials; if unaddressed, these may lead to a decreased probability of success (10). Some of these challenges can be mapped to an incomplete mechanistic understanding of immune response dynamics and the interplay of such immune responses with tumor infiltration processes and tumor cell growth (11). These quantitative knowledge gaps hinder: (i) effective translation of novel promising therapeutic approaches into the clinic, (ii) identification of predictive response biomarkers, and (iii) search of therapeutic drug combinations which may overcome intrinsic or acquired resistance to existing standards of care (12). This mini-review focuses on quantitative, mechanistically-oriented modeling approaches which have been sought in IO, to address, at least partially, the abovementioned challenges and knowledge gaps.

## Evolution of Quantitative, Mechanistically-Oriented IO Systems Modeling

**Figure 1:**
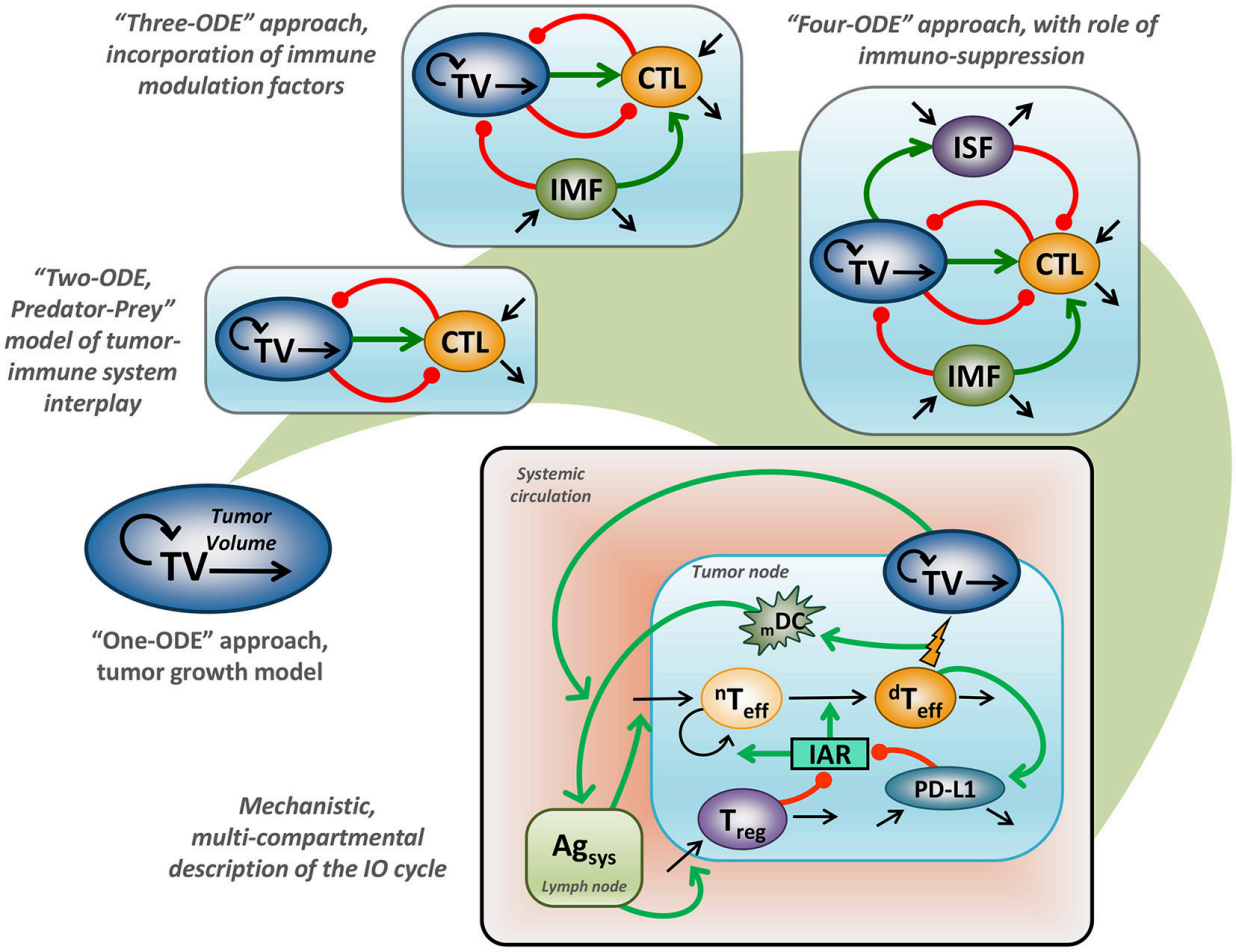
Evaluation of mathematical models which describe tumor vs. immune system interactions. “One-ODE” approach, simplistic description of tumor growth kinetics; “Two-ODE” approach, a typical “Predator-Prey” model, incorporating a basic description of tumor vs. immune system interactions; “Three-ODE” approach, incorporating additional immuno-modulating factor(s); “Four-ODE” approach, including considerations for immuno-suppression; mechanistic multi-compartmental model, taking into account essential biological principles underlying the IO cycle concept (21); TV, tumor volume; CTL, cytotoxic T lymphocytes; IMF, immuno-modulating factor; ISF, immuno-suppressive factor; mDC, level of mature dendritic cells; _n_T_eff_, non-differentiated T effectors cells; _d_T_eff_, differentiated T effectors cells; Treg, regulatory T cells; PD-L1, level of PD-L1 expression; Ag_sys_, level of systemic antigen; IAR, immuno-activation rate function; green line, positive regulation, red line negative, regulation; back line, variable turnover.

Application of mathematical modeling in support of preclinical and clinical research, as well as decision-making in Oncology, has a long-standing history covering multiple problems and addressing a variety of research questions—today often referred to as computational oncology (13–15). Historical milestones include adaptations of the Gompertz model for treatment outcomes in breast cancer (16). These earlier efforts started from models with a simplistic empirical structure, based on an ordinary differential equation (ODE) describing tumor size growth using an exponential or sigmoidal function (17). Such a model, however, would not adequately describe the interplay between tumor cells and tissue vs. the immune system, since it entirely ignores the immune component (18). It is nevertheless valuable to mathematically describe treatment response effects following various chemotherapies, which are adequately captured by generalized Gompertzian kinetics (19). In fact, such modeling results provided a basis for the use of specific “dose-dense” chemotherapeutic regimens, which subsequently showed favorable outcomes in the treatment of breast cancer (20). Additionally, such empirical considerations allowed for a gradual evolution of modeling concepts, which today can be grounded in mechanistically-oriented principles, including for tumor *vs*. immune system interactions (Figure 1).

Earlier efforts to describe tumor vs. immune system relationships via a general mathematical description appeared in the 1980's, following the pioneering IO work that introduced the concept of immune surveillance (2, 3). These mathematical models considered the addition of a second variable describing the dynamics of cytotoxic immune cells, which are able to attack tumor cells (22–24). The resultant “two-ODE” model actually follows a typical “predator-prey” model introduced by Alfred Lotka and Vito Volterra, in much earlier days, at the turn of the 20th century. In such a model, tumor cells may be interpreted as the “prey,” whereas cytotoxic immune cells may be viewed as the “predator”: their dynamic interplay may result in one possible system behavior reflective of cancer dormancy (4). Given the relative simplicity of such a “two-ODE” model and since the behavior of such a model could be assessed analytically, it gained immense popularity within the oncology modeling community and led to several theoretical hypotheses underlying fundamental principles of cancer progression. For example, it was shown, through modeling, that key parameters controlling tumor re-growth under steady-state conditions of cancer dormancy were those relating to activities of the immune system (25). A corollary result was that it is a reduction in the probability of achieving tumor cell kill, rather than a reduction in the probability of tumor cells being recognized by cytotoxic cells, which best explained immune evasion by tumor cells (26). Interestingly, this key result, derived theoretically at the time, has recently been supported by elegant modeling work linking high-level immunological and epidemiological data, which suggests that age-related decline in T cell output correlates better with risk of cancer diagnosis vs. age-related accumulation of somatic mutations in tumor cells (27).

With the explosive growth of experimental data surrounding the complexity of tumor vs. immune system interplay, “two-ODE” models experienced a further evolution with additional biological entities and mechanisms being taken into mathematical consideration. At this point and looking forward, many biological candidates were tested as the “third modeling variable,” representing either specific immune cells or cytokines that modulate cytotoxic T lymphocyte (CTL) function (28). Such models were initially focused on including IL-2 function and effects, reflective of the potential importance of this cytokine and its associated dynamics in long-term tumor relapse (29). In further work, de Pillis et al. used a “three-ODE” model to reveal a difference between the dynamics of CD8^+^ CTLs *vs*. natural killer cells, which supported the importance of considering multiple cell types in the overall anti-tumor immune activity (30). More recently, CD4^+^ T helper cells were considered as the third component, in a quantitative, model-based investigation of adoptive cellular immunotherapy (31).

“Three-ODE” models, however, exhibit one significant structural limitation, namely they completely lack (an) immuno-suppressive component(s), which would be crucial when considering immune evasion mechanisms (32). Therefore, embedding a fourth variable into such models, to describe immuno-suppression, would seem rather natural; however, choices for the most appropriate candidate in this role are multi-fold. Several types of immuno-suppressive cells or molecules could be suitable candidates, including regulatory T cells (Tregs), myeloid-derived suppressor cells (MDSCs), or Type 2 tumor-associated macrophages, as well as cytokines such as TGFβ or IL-10. Thus, Arciero et al. chose TGFβ as the fourth model variable (33), while de Pillis et al. used Tregs as the principal immuno-suppressive component in their model (34). While these two modeling examples focused on immuno-suppressive effectors, other “four-ODE” models abound, declining a vast variety of immune players or tumor cell clones (35–41). Such a variety in potential key immuno-modulating factors made the generalization of any “three-ODE” or “four-ODE” model an overly difficult process, since any one of the models cited above can be challenged with newly generated experimental data featuring the importance of one vs. another immune factor. This may also explain, at least partially, the relatively minimal recognition, to date, of quantitative modeling approaches by immuno-oncologists (28, 42, 43).

On one hand, some of the biological complexities which compose the IO cycle, as summarized in recent reviews (6, 44, 45) clearly indicate the limitations of oversimplified models such as “prey-predator” models, which appear to be too remote from experimental reality and would not be applicable or of use for the majority of research relevant questions. On the other hand, increasing model complexity with additional mechanistic insights always comes with challenges of model calibration, as depicted in this famous quote by John von Neumann, “*with four parameters, I can fit an elephant and with five, I can make him wiggle his trunk*” [see in Dyson (46)]—pointing to the necessity of avoiding overparameterized “metastatic” models with unreliable extensions and loss of predictive power. Achieving such a balance in capturing necessary (not over-simplified) yet sufficient (not over-developed) features, and as constrained by the available data, is arguably one of the most difficult challenges in fit-for-purpose, parsimonious mechanistic model building and calibration. Overparameterization can easily negate all benefits brought forward by the incorporation of exquisite biological details of the system under consideration (47); models which attempt to explain everything may in fact not be useful, their predictive power remaining a question mark (48).

To address this challenge, part of the solution may reside in the combining of modeling methodologies developed previously and in other disciplines (49). This would result in a repository of prior information and knowledge validated elsewhere, to build mechanistic models in immuno-oncology which, on one hand, incorporate increasing system complexity and, on the other hand, avoid overparameterization based on newly generated data—thereby resulting, using terminology of a Bayesian mindset, in a posterior model based on existing, established quantitative priors (50).

If so, the question then becomes, “where to find such established prior models?” One obvious domain is quantitative immunology (51, 52), where the use of various modeling techniques by experimentalists has already gotten significantly more traction, arguably, than in other fundamental biological disciplines (53). For example, modeling has provided quantitative “inference frameworks” for immunology basics and fundamentals such as T cell activation, homeostasis or self / non-self recognition (54–57), immune receptor signaling (58), and understanding of T cell immunological memory (59). Prior models from quantitative immunology may then be combined with prior models from quantitative pharmacology (60–62), another field where modeling has provided quantitative “inference frameworks” (63) In the next section, we will discuss selected works which considered the combining of modeling methodologies, in attempts to develop pharmacologically-modulated posterior models, which were then used to prospectively address questions in the development of IO therapies (Table 1).

**Table 1:** Mechanistic models in support of IO therapy development.

**Description^**a**^**	**Application^**a**^**	**Limitations^**a**^**	**References**
Cergutuzumab amunaleukin (CEA mAb-IL2v fusion protein) PK/PD described using a population NLME modeling approach	Model was used to identify optimal dosing regimen and support design of the clinical dose escalation study	Since mechanisms of tumor vs. immune system interactions have not been considered, the model cannot be generalized to other MoAs nor their combinations	(64)
Pembrolisumab (αPD-1 mAb) PK/PD described using a population NLME modeling approach	Model was used to estimate MABEL dose and was applied, accordingly, for FIH dose selection	1. Since mechanisms of tumor vs. immune system interactions have not been considered, the model cannot be generalized to other MoAs nor their combinations 2. Model is based on preclinical data only	(65)
DART against CD3 and P-cadherin PK/PD was described using a simple ODE modeling framework	1. Model was used to estimate MABEL dose and was applied, accordingly, for FIH dose selection 2. Model was further applied for identification/better characterization of PK/PD relationship and MoA	1. Since mechanisms of tumor vs. immune system interactions have not been considered, the model cannot be generalized to other MoAs nor their combinations 2. Model is based on preclinical data only and does not take into account variability	(66)
Multiple MoAs including vaccination (CyaA-E7), TLR9 agonist (CpG), chemotherapy (cyclophosphamide), and IL-12 administration were incorporated using a NLME modeling approach	Model was applied for a better understanding of synergistic effects in combination treatment	1. Due to the simple description of tumor vs. immune system interactions, the model cannot be generalized to other MoAs 2. Model is based on preclinical data only	(21, 67)
Multiple MoAs including αPD-1 and αPD-L1, αCTLA4 mAb, OX40 agonists, CXCR2 inhibitors, and RT were incorporated using a population NLME modeling approach	1. Model was applied for a better understanding of synergistic effects in combination treatment and identification of predictive biomarkers 2. Based on the model simulations, an optimal sequencing schedule was proposed for the combination treatment	1. Model is based on preclinical data only	(68, 69)
RT and αCTLA4 mAb were described using a simple ODE modeling framework	Model was applied to guide optimal combination treatment doses and schedules	1. Due to the simple description of tumor vs. immune system interactions, pharmacological interventions and limited validation with experimental data, the model cannot be generalized to other MoAs nor used for clinically relevant simulations 2. Model is based on preclinical data only and does not take into account variability	(70)
Mechanistic physiologically-based description of clinically-relevant immune cell fluxes and RT	1. Model was applied for a better understanding of ICD systemic effects 2. Optimal RT administration sites for metastatic solid tumors were identified	1. Limited validation with clinical data was performed during model development stage 2. Model does not take into account variability	(71)
Multiple MoAs including αPD-L1, BRAF and MEK inhibitors and vaccination (GVAX) were incorporated using a simple PDE modeling framework, to account for spatial immune species distribution within the tumor compartment	Model was applied for a better understanding of synergistic effects	1. Due to the simple description of tumor vs. immune system interactions, pharmacological interventions and limited validation with experimental data, the model cannot be generalized to other MoAs nor used for clinically relevant simulations 2. Model is based on preclinical data only and does not take into account variability	(72, 73)
Multiple MoAs including vaccination (UV-8101-RE), IL-2 neutralization, Treg cell depletion, androgen deprivation therapy and castration were incorporated using a simple ODE modeling framework	Model was applied to guide optimal combination treatment schemes	1. Due to the simple description of tumor vs. immune system interactions, pharmacological interventions and limited validation with experimental data, the model cannot be generalized to other MoAs nor used for clinically relevant simulations; 2. Model is based on the preclinical data only and does not take into account variability	(74)
Alloreactive cytotoxic-T-lymphocytes transfer was described using a simple ODE modeling framework	Model was applied for the identification of predictive biomarkers	1. Model does not take into account variability 2. Limited validation with clinical data was performed	(75)
IL-21 administration was described using a simple ODE modeling framework	Model was applied for a better MoA understanding and the identification of predictive biomarkers	1. Model is based on preclinical data only and does not take into account variability 2. Limited validation with clinical data was performed	(76)
Prostate cancer vaccination effects were described using a simple ODE modeling framework	Model was applied for an evaluation of personalized treatment strategies	1. Limited validation with clinical data was performed	(77)
Multiple MoAs including αPD-L1 mAb, BTK inhibitor (ibrutinib), and vaccination were incorporated using a simple ODE modeling framework	Model was applied for guiding optimal combination treatment schemes	1. Due to the simple description of tumor vs. immune system interactions, pharmacological interventions and limited validation with experimental data model cannot be generalized to other MoAs nor used for clinically relevant simulations; 2. Model is based on preclinical data only and does not take into account variability	(78)
αPD-L1 mAb clinical effects were described using a 3D ABM framework	Model was applied for an evaluation of personalized treatment strategies	1. Limited validation with clinical was performed 2. Systemic treatment effects were not considered	(79)
Generalized effects of adaptive immunity stimulation and stromal cell depletion were described using a 2D and 3D ABM framework	Model was applied for guiding optimal combination treatment schemes	1. Generic representation of treatment effects	(80, 81)

a *MoA, Mechanism of action; CEA, carcinoembryonic antigen; mAb, monoclonal antibody; NLME, nonlinear mixed effects; IO, immuno-oncology; PK, pharmacokinetics; PD, pharmacodynamics; MABEL, minimally anticipated biological effect level; FIH, first-in-human; RT, radiotherapy; ICD, immunologic cell death; ODE, ordinary differential equations; PDE, partial differential equation; ABM, agent-based modeling*.

## Mechanistic Modeling in Support of IO Therapy Development

Applications of mechanistic modeling in support of preclinical and clinical research, commonly referred to as pharmacokinetic (PK)/pharmacodynamic (PD) modeling, are traditionally centered around the optimization of treatment dosing and scheduling—the “dose” representing a critical component of any drug development program (82). Such modeling approaches have thus been used in the development of IO agents such as PD-1 and PD-L1 inhibitors (83–85). In particular, mechanistic PKPD modeling has been applied in support of first-in-human dose selection of pembrolizumab, an anti PD-1 agent (65); this resulted in a seamless clinical trial design with a model-informed dose justification, which the US FDA accepted in the process of an accelerated regulatory review (86). Label updates with flat dosing schedules were subsequently granted, for both nivolumab and pembrolizumab, strongly supported by model-based simulations (87, 88). PKPD modeling has also been used in the translation of preclinical data for a conjugated IL-2 therapy, in particular to gain a better understanding of such a therapy's downstream effects (89). PKPD modeling has been further used in the development of bispecific biologics. Chen et al. used it for the estimation of the minimally anticipated biological effect level (MABEL) of a bispecific antibody targeting CD3 and p-cadherin (66), while Ribba et al. used it for guided dose escalation study design of cergutuzumab amunaleukin, a fusion protein consisting of IL-2 and a carcinoembryonic antigen (CEA) human monoclonal antibody (64). Such models are great examples of a “fit-for-purpose” quantitative approach, focused on addressing a specific pharmacological question. However, they do not take into account details of the tumor vs. immune system interactions, which would be critical to gain a better understanding of mechanisms of action (MoA) of immunotherapies.

Progressively adding components of tumor vs. immune system interactions into such PKPD models may well support the addressing of questions around pharmacologically-modulated IO biology, a topic of paramount importance in, for example, the search for therapeutic IO drug combinations (90). Such a systems approach may become an indispensable quantitative tool supporting “go/no-go” decisions in development programs, especially if sufficient biological knowledge for viable generalization is considered in the model (91). This prior knowledge is generally derived from two sources: (i) connectivity information to determine the system structure, e.g., molecular & cellular interactions, and their integration into patho-physiological processes; and (ii) quantitative data, for the calibration of model parameters. As discussed in the previous section, an imbalance in structural *vs*. quantitative information will in one way or another complicate integration into, and practical use of a mathematical model. For example, Lai and Friedman developed an elegant, yet complex model which includes a high number of biological elements, and considered their dynamics in space and time using partial differential equations (PDEs), to better understand the potential synergy between PD-(L)1 antagonists and either a GVAX vaccination or BRAFi/MEKi targeted therapies (72, 73). However, assessing the predictive power of such a model is impractical, given insufficient experimental data for model validation. Serre et al. provided another example of an elegant, yet insufficiently validated mathematical model describing the potential synergy between radiotherapy (RT) and immune checkpoint blockade (70).

One obvious way to improve model validation and hence model predictive power is to use rich experimental data, to rigorously constrain model parameters. This, however, requires the use of adequate statistical methods to properly quantify uncertainty and variability, which are inherent to any experimental biomedical and life sciences dataset (49, 92). In oncology drug development, quantitative data supporting MoA elucidation are typically generated at the preclinical stage. Parra-Guillen et al. for example, used a nonlinear mixed-effects (NLME) model and experimental data from syngeneic tumor models, to reveal the most influential immuno-adjuvant capable of boosting anti-tumor vaccination effects (21, 67). Such a modeling approach, which combines mechanistic features and mixed effects, allows one to incorporate individual-level data into the model, which may then describe not only mean trends, but also the full range of individual biomarker dynamics (93). A similar, combined mechanistic and mixed-effects approach was used to develop a model describing synergistic effects between RT and PD-(L)1 blockade in mice (68). This model, in fact, synthesizes a fit-for-purpose, yet sufficiently detailed mathematical description of the IO cycle, together with adequate model validation based on data from multiple experiments. As a result, this model can be used as a simulation tool for experimental study design, and is also adequate for determining optimal schedule and sequencing of RT + IO, and IO + IO treatment combinations (68, 69). Interestingly, despite the well-known challenges in translating oncology preclinical results into the clinic, simulation results from this preclinical modeling exercise were recently supported, in a qualitative sense, with clinical data and a corresponding meta-analysis (94, 95). For a quantitative translation, the Kosinsky et al. model would require adjustments for multiple quantitative differences that exist between mouse *vs*. human immune systems, e.g., appropriate expressions of immune checkpoints and turnover of specific T cells (96). Another modeling approach aimed at supporting the development of such an RT + IO combination therapy was proposed by Poleszczuk et al. who developed a physiologically-based model which considered a detailed incorporation of T cell trafficking and was used for the identification of an optimal site for RT administration, to maximally increase the probability of incremental anti-tumor immune effects (71). Predictions from such a comprehensive modeling effort were also recently supported by clinical results, which showed that RT administered to liver metastases triggered a higher immunological response (97). A mechanistic model has also been proposed by Peng et al. in the search of an optimal combination strategy against castration-resistant prostate cancer (74).

The modeling applications discussed to this point emphasize the importance of addressing multi-pronged questions, e.g., not only around dose finding, but also on the identification of an adequate time window for maximizing therapeutic benefits (98). This problem is particularly challenging in the development of combination therapies, where multiple options around which cancer indication, which combination agents, which scheduling per agent, and which sequencing of the agents make trial design enormously complex (99, 100). In recent years, platform design of clinical studies, driven by one master protocol, has gained momentum (101, 102)—a format which, in fact, benefits even further from a supportive quantitative mechanistic modeling approach (103).

## Mechanistic Modeling in Support of IO Biomarker Identification

A third problem which is highly relevant in the development of IO therapies is the identification of predictive biomarkers. Indeed, there still is a lot of room for improving numbers of responder patients in pivotal IO trials, even in immunologically-active indications (104). Several computational models focusing on the identification of predictive biomarkers, with applications to personalized treatment against glioblastoma and prostate cancer have been developed (75, 77). These approaches have yet to find a general use in clinical practice. Part of the challenge arises from the biological complexity in the IO field, although there also are significant limitations from an experimental standpoint, such as differences in fresh vs. archived samples, difficulties in obtaining multiple biopsies per patient, with related risk and cost issues (105). One approach to alleviate some of these problems is the development of novel combinatorial biomarkers (“signatures”) which may relate multiple, routinely measured markers with clinically meaningful biological phenotypes (106). In fact, such a consensus approach, “Immunoscore,” has recently been validated in a large international study of colon cancer (107).

Another complicating factor in the development and interpretation of mechanistic modeling of IO data is the tremendous heterogeneity in tumor cell clones and elements of the surrounding immune microenvironment (108). A rapid development of novel experimental techniques may overcome this challenge, at least partially. Thus, the identification of specific gene expression signatures may help in further validating existing immunoscores and related biomarkers, even increasing their discriminatory ability (109), as recently shown with a PD-L1 expression signature which outperformed a standard PD-L1 immunohistochemistry (IHC) assay (110). Multiple immune signatures have now been identified, which allow for a better characterization of various aspects of anti-tumor immunity (111–116). Recent technological breakthroughs such as cytometry by time-of-flight (CyTOF) and single-cell mRNA sequencing (scRNA-seq) may further advance the utility and robustness of these immune signatures (117, 118); these techniques may allow for a deeper, more granular profiling of tumor and immune cell phenotypes involved in response or resistance to immunotherapies, in multiple indications (119–123). The importance in using quantitative models toward the selection and qualification (within the chain of events, from dosing to patient response) of IO biomarker signatures cannot be over-emphasized (108): immune biomarkers involve a high number of molecular and cellular species, and often exhibit complex temporal and spatial dynamics; these need to be properly framed in the context of a quantitative model, especially if the purpose is to relate multi-variate biomarker signatures to IO treatment effects and clinical endpoints (124). Quantitative modeling may also support the development of biomarkers in context, by integrating different data types, and following a model-based qualification of biomarkers as surrogate measures of efficacy and response. Such an approach has been proposed, recently, in the evaluation of neoantigen fitness as a surrogate measure of immunogenic quality of the existing neoantigen pool (125, 126). The progressive integration of such consensus, multi-variate combinatorial biomarkers into a unified, quantitative and mechanistic modeling framework will help overcome some of the limitations in the clinical use of IO biomarkers (127, 128).

## Other Mechanistic Modeling Approaches With Relevance to IO

The above sections focused on traditional deterministic models, which make use of ODEs and PDEs for the description of IO systems dynamics. Other modeling techniques can be used to describe tumor *vs*. immune interactions. For example, cellular automata and agent-based models (ABMs) (129), as well as various hybrid models which link continuous and discrete modeling elements have been developed (130). Such models may be useful in raising new hypotheses, which may arise from emergent properties of the system based on existing data, rather than generating *bona fide* forward predictions. For example, a lattice gas automata technique has been used to gain a better understanding of a vaccination treatment mechanism and its corresponding anti-tumor immune response dynamics (131, 132). ABMs also represent a popular modeling technique, since they are well-suited to describe stochastic processes which do occur at various stages of the IO cycle. For example, Gong et al. developed an ABM to reveal spatio-temporal characteristics of PD-L1 blockade (79). In another publication, Kather et al. presented an elegant 2D ABM framework for an improved understanding of the role of stromal cells in colorectal cancer (CRC) (80). These authors determined that malignant cells hiding in the stroma cannot be eradicated completely, while stromal cells, at the same time, would not allow for rapid tumor progression. Consequently, simulations of an immuno-therapy illustrated how stroma permeabilization, concomitantly with immune activation, were able to markedly increase response to therapy *in silico*. Additionally, it was shown that a stroma-targeted therapy with insufficient activation of tumor-specific CTLs can lead to rapid tumor escape and hyper-progression (80). More recently, this model has been extended and generalized to a 3D spatial description, incorporating macrophage effects; it accurately reproduced the tissue architecture typically observed in CRC and can be used, similarly to ODE systems models, for the identification of effective IO therapeutic combinations (81).

## Concluding Remarks

Following the approval, in recent years, of the first immune checkpoint inhibitors, the landscape of cancer treatment has changed dramatically and has shifted to a deep reconsideration of the role of the immune system in cancer progression and treatment. This led to an unprecedented number of clinical trials and generation of clinical data in the IO field. Clinical success rates, however, while improving significantly, are still relatively low. The observed imbalance, between the amount of biological and clinical data being generated *vs*. probability of trial success is not uncommon in biomedical disciplines, and calls for the development and updating of a companion, integrative, quantitative modeling framework with predictive value for MoAs and simulation value for study design purposes. As described by Sidney Brenner in his “*Sequences and Consequences*” landmark paper: “*We should welcome with open arms everything that modern technology has to offer us but we must learn to use it in new ways. Biology urgently needs a theoretical basis to unify it and it is only theory that will allow us to convert data to knowledge*” (133). We propose that quantitative, mechanistically-oriented modeling represents a means toward the establishment of such a “theoretical basis,” pending proper integration of prior knowledge gained from biology and clinical research. One of the main factors limiting a wider application of quantitative systems modeling is its demand for rich experimental data necessary for precise parameter estimation. Historically, generation of such datasets in oncology research has been challenging, due to translational limitations of experimental preclinical models and sparse collection of tissue samples in clinical settings. Also, in the IO field, another challenge is the lack of predictive power for univariate biomarkers (e.g., PD-L1 IHC status or tumor mutational burden taken in isolation), which may unequivocally link immunologically-driven therapeutic effects to clinical response; a multi-variate approach is clearly needed (128). Recent developments in multi-modality biomarkers and associated molecular signatures, together with innovative pharmacologies and clinical design under platform trials (134) will help in the progressive build-out and qualification of such a unified quantitative modeling framework, which in turn may help in predicting patient responses based on a given pharmacological intervention choice and multi-variate biomarker signatures.

## Author Contributions

KP, VV, and YK generated Figure 1. The Table was generated by IA, VV, and LC. All authors contributed to the writing of the manuscript.

### Conflict of Interest Statement

The authors declare that this study received funding from AstraZeneca. VV, IA, KP, and YK are employed and KP, YK are owners of M&S Decisions, a modeling consultancy received research funding from AstraZeneca. LC and GH are employed by, and GH is a shareholder of AstraZeneca. The authors declare that the research was conducted in the absence of any commercial or financial relationships that could be construed as a potential conflict of interest.

## References

[1] Coley WB. The treatment of inoperable sarcoma by bacterial toxins (the mixed toxins of the Streptococcus erysipelas and the Bacillus prodigiosus). Proc R Soc Med. (1910) 3:1–48. 19974799 10.1177/003591571000301601 PMC1961042

[2] Ehrlich P Collected Papers of Paul Ehrlich: in Four Volumes Including a Complete Bibliography. London : Pergamon Press (1956).

[3] Burnet FM. Immunological surveillance in neoplasia. Transplant Rev. (1971) 7:3–25. 5146537 10.1111/j.1600-065x.1971.tb00461.x

[4] Uhr JW Scheuermann RH Street NE Vitetta ES. Cancer dormancy: opportunities for new therapeutic approaches. Nat Med. (1997) 3:505–9. 10.1038/nm0597-505 9142117

[5] Dunn GP Bruce AT Ikeda H Old LJ Schreiber RD. Cancer immunoediting: from immunosurveillance to tumor escape. Nat Immunol. (2002) 3:991–8. 10.1038/ni1102-991 12407406

[6] Chen DS Mellman I. Oncology meets immunology: the cancer-immunity cycle. Immunity. (2013) 39:1–10. 10.1016/j.immuni.2013.07.012 23890059

[7] Tang J Shalabi A Hubbard-Lucey VM. Comprehensive analysis of the clinical immuno-oncology landscape. Ann Oncol. (2018) 29:84–91. 10.1093/annonc/mdx755 29228097

[8] Tang J Yu JX Hubbard-Lucey VM Neftelinov ST Hodge JP Lin Y. Trial watch: the clinical trial landscape for PD1/PDL1 immune checkpoint inhibitors. Nat Rev Drug Discov. (2018) 17:854–5. 10.1038/nrd.2018.210 30482962

[9] Fox BA Schendel DJ Butterfield LH Aamdal S Allison JP Ascierto P. Defining the critical hurdles in cancer immunotherapy. J Transl Med. (2011) 9:214. 10.1186/1479-5876-9-214 22168571 PMC3338100

[10] Baik CS Rubin EH Forde PM Mehnert JM Collyar D Butler MO. Immuno-oncology clinical trial design: limitations, challenges, and opportunities. Clin Cancer Res. (2017) 23:4992–5002. 10.1158/1078-0432.CCR-16-3066 28864727 PMC5735832

[11] Klinke DJ. Enhancing the discovery and development of immunotherapies for cancer using quantitative and systems pharmacology: interleukin-12 as a case study. J Immunother Cancer. (2015) 3:27. 10.1186/s40425-015-0069-x 26082838 PMC4468964

[12] Sharma P Hu-Lieskovan S Wargo JA Ribas A. Primary, adaptive, and acquired resistance to cancer immunotherapy. Cell. (2017) 168:707–23. 10.1016/j.cell.2017.01.017 28187290 PMC5391692

[13] Gallasch R Efremova M Charoentong P Hackl H Trajanoski Z. Mathematical models for translational and clinical oncology. J Clin Bioinforma. (2013) 3:23. 10.1186/2043-9113-3-23 24195863 PMC3828625

[14] Altrock PM Liu LL Michor F. The mathematics of cancer: integrating quantitative models. Nat Rev Cancer. (2015) 15:730–45. 10.1038/nrc4029 26597528

[15] Barbolosi D Ciccolini J Lacarelle B Barlési F André N. Computational oncology–mathematical modelling of drug regimens for precision medicine. Nat Rev Clin Oncol. (2016) 13:242–54. 10.1038/nrclinonc.2015.204 26598946

[16] Norton L Simon R Brereton HD Bogden AE. Predicting the course of Gompertzian growth. Nature. (1976) 264:542–5. 1004590 10.1038/264542a0

[17] Araujo R. A history of the study of solid tumour growth: the contribution of mathematical modelling. Bull Math Biol. (2004) 66:1039–91. 10.1016/j.bulm.2003.11.002 15294418

[18] Agur Z. From the evolution of toxin resistance to virtual clinical trials: the role of mathematical models in oncology. Future Oncol. (2010) 6:917–27. 10.2217/fon.10.61 20528230

[19] Norton L. A Gompertzian model of human breast cancer growth. Cancer Res. (1988) 48:7067–71. 3191483

[20] Norton L. Conceptual and practical implications of breast tissue geometry: toward a more effective, less toxic therapy. Oncologist. (2005) 10:370–81. 10.1634/theoncologist.10-6-370 15967831

[21] Parra-Guillen ZP Berraondo P Ribba B Troconiz IF. Modeling tumor response after combined administration of different immune-stimulatory agents. J Pharmacol Exp Ther. (2013) 346:432–42. 10.1124/jpet.113.206961 23845890

[22] Stepanova NV Course of the immune reaction during the development of a malignant tumor. Biophysics. (1980) 24:917–23.

[23] Kuznetsov VA. A mathematical model for the interaction between cytotoxic T lymphocytes and tumour cells. Analysis of the growth, stabilization, and regression of a B-cell lymphoma in mice chimeric with respect to the major histocompatibility complex. Biomed Sci. (1991) 2:465–76. 1840834

[24] Kuznetsov V Makalkin I Taylor M Perelson A. Nonlinear dynamics of immunogenic tumors: parameter estimation and global bifurcation analysis. Bull Math Biol. (1994) 56:295–321. 10.1016/S0092-8240(05)80260-5 8186756

[25] Kuznetsov VA Knott GD Modeling tumor regrowth and immunotherapy. Math Comput Model. (2001) 33:1275–87. 10.1016/S0895-7177(00)00314-9

[26] d'Onofrio A Ciancio A. Simple biophysical model of tumor evasion from immune system control. Phys Rev E. (2011) 84:031910. 10.1103/PhysRevE.84.031910 22060406

[27] Palmer S Albergante L Blackburn CC Newman TJ. Thymic involution and rising disease incidence with age. Proc Natl Acad Sci USA. (2018) 115:1883–8. 10.1073/pnas.1714478115 29432166 PMC5828591

[28] Eftimie R Bramson JL Earn DJD. Interactions between the immune system and cancer: a brief review of non-spatial mathematical models. Bull Math Biol. (2011) 73:2–32. 10.1007/s11538-010-9526-3 20225137

[29] Kirschner D Panetta JC. Modeling immunotherapy of the tumor-immune interaction. J Math Biol. (1998) 37:235–52. 9785481 10.1007/s002850050127

[30] de Pillis LG Radunskaya AE Wiseman CL A validated mathematical model of cell-mediated immune response to tumor growth. Cancer Res. (2005) 65:7950–8. 10.1158/0008-5472.CAN-05-0564 16140967

[31] Dong Y Miyazaki R Takeuchi Y Mathematical modeling on helper T cells in a tumor immune system. Discrete Contin Dyn Syst - Ser B. (2013) 19:55–72. 10.3934/dcdsb.2014.19.55

[32] Zindl CL Chaplin DD. Tumor immune evasion. Science. (2010) 328:697–8. 10.1126/science.1190310 20448171 PMC4123868

[33] Arciero JC Jackson TL Kirschner DE A mathematical model of tumor-immune evasion and siRNA treatment. Discrete Contin Dyn Syst - Ser B. (2003) 4:39–58. 10.3934/dcdsb.2004.4.39

[34] dePillis L Caldwell T Sarapata E Williams H Mathematical modeling of regulatory T cell effects on renal cell carcinoma treatment. Discrete Contin Dyn Syst - Ser B. (2013) 18:915–943. 10.3934/dcdsb.2013.18.915

[35] Nani F Freedman HI. A mathematical model of cancer treatment by immunotherapy. Math Biosci. (2000) 163:159–99. 10.1016/S0025-5564(99)00058-9 10701303

[36] Szymanska Z Analysis of immunotherapy models in the context of cancer dynamics. Int J Appl Math Comput Sci. (2008) 13:407–18.

[37] Villasana M Radunskaya A. A delay differential equation model for tumor growth. J Math Biol. (2003) 47:270–94. 10.1007/s00285-003-0211-0 12955460

[38] Byrne HM Cox SM Kelly CE Macrophage-tumour interactions: *in vivo* dynamics. Discrete Contin Dyn Syst - Ser B. (2003) 4:81–98. 10.3934/dcdsb.2004.4.81

[39] Bunimovich-Mendrazitsky S Shochat E Stone L. Mathematical model of BCG immunotherapy in superficial bladder cancer. Bull Math Biol. (2007) 69:1847–70. 10.1007/s11538-007-9195-z 17457655

[40] Wilson S Levy D. A mathematical model of the enhancement of tumor vaccine efficacy by immunotherapy. Bull Math Biol. (2012) 74:1485–500. 10.1007/s11538-012-9722-4 22438084 PMC3822329

[41] Nanda S dePillis L Radunskaya A B cell chronic lymphocytic leukemia - a model with immune response. Discrete Contin Dyn Syst - Ser B. (2013) 18:1053–76. 10.3934/dcdsb.2013.18.1053

[42] Walker R Enderling H. From concept to clinic: mathematically informed immunotherapy. Curr Probl Cancer. (2016) 40:68–83. 10.1016/j.currproblcancer.2015.10.004 26645497

[43] dePillis LG Eladdadi A Radunskaya AE. Modeling cancer-immune responses to therapy. J Pharmacokinet Pharmacodyn. (2014) 41:461–78. 10.1007/s10928-014-9386-9 25281420

[44] Mellman I Coukos G Dranoff G. Cancer immunotherapy comes of age. Nature. (2011) 480:480–9. 10.1038/nature10673 22193102 PMC3967235

[45] Chen DS Mellman I. Elements of cancer immunity and the cancer–immune set point. Nature. (2017) 541:321–30. 10.1038/nature21349 28102259

[46] Dyson F. A meeting with enrico fermi. Nature. (2004) 427:297. 10.1038/427297a 14737148

[47] Azeloglu EU Iyengar R. Good practices for building dynamical models in systems biology. Sci Signal. (2015) 8:fs8. 10.1126/scisignal.aab0880 25852187

[48] Gunawardena J. Models in biology: “accurate descriptions of our pathetic thinking.” BMC Biol. (2014) 12:29. 10.1186/1741-7007-12-29 24886484 PMC4005397

[49] Tsigkinopoulou A Baker SM Breitling R. Respectful modeling: addressing uncertainty in dynamic system models for molecular biology. Trends Biotechnol. (2017) 35:518–29. 10.1016/j.tibtech.2016.12.008 28094080

[50] Tarantola A Popper, Bayes, and the inverse problem. Nat Phys. (2006) 2:492–4. 10.1038/nphys375

[51] Andrew SM Baker CTH Bocharov GA Rival approaches to mathematical modelling in immunology. J Comput Appl Math. (2007) 205:669–86. 10.1016/j.cam.2006.03.035

[52] Ludewig B Stein JV Sharpe J Cervantes-Barragan L Thiel V Bocharov G A global “imaging” view on systems approaches in immunology: HIGHLIGHTS. Eur J Immunol. (2012) 42:3116–25. 10.1002/eji.201242508 23255008

[53] Louzoun Y. The evolution of mathematical immunology. Immunol Rev. (2007) 216:9–20. 10.1111/j.1600-065X.2006.00495.x 17367331

[54] Grossman Z Paul WE. Autoreactivity, dynamic tuning and selectivity. Curr Opin Immunol. (2001) 13:687–98. 10.1016/S0952-7915(01)00280-1 11677091

[55] Grossman Z. Mathematical modeling of thymopoiesis in HIV infection: real data, virtual data, and data interpretation. Clin Immunol. (2003) 107:137–9. 10.1016/S1521-6616(03)00122-0 12804526

[56] Grossman Z Min B Meier-Schellersheim M Paul WE. Concomitant regulation of T-cell activation and homeostasis. Nat Rev Immunol. (2004) 4:387–95. 10.1038/nri1355 15122204

[57] Khailaie S Bahrami F Janahmadi M Milanez-Almeida P Huehn J Meyer-Hermann M. A mathematical model of immune activation with a unified self-nonself concept. Front Immunol. (2013) 4:474. 10.3389/fimmu.2013.00474 24409179 PMC3872974

[58] Goldstein B Faeder JR Hlavacek WS. Mathematical and computational models of immune-receptor signalling. Nat Rev Immunol. (2004) 4:445–56. 10.1038/nri1374 15173833

[59] Antia R Ganusov VV Ahmed R. The role of models in understanding CD8+ T-cell memory. Nat Rev Immunol. (2005) 5:101–11. 10.1038/nri1550 15662368

[60] Danhof M. Systems pharmacology – towards the modeling of network interactions. Eur J Pharm Sci. (2016) 94:4–14. 10.1016/j.ejps.2016.04.027 27131606

[61] Helmlinger G Al-Huniti N Aksenov S Peskov K Hallow KM Chu L Drug-disease modeling in the pharmaceutical industry - where mechanistic systems pharmacology and statistical pharmacometrics meet. Eur J Pharm Sci. (2017) 109:S39–46. 10.1016/j.ejps.2017.05.028 28506868

[62] Musante C Ramanujan S Schmidt B Ghobrial O Lu J Heatherington A. Quantitative systems pharmacology: a case for disease models. Clin Pharmacol Ther. (2017) 101:24–7. 10.1002/cpt.528 27709613 PMC5217891

[63] Black J. A personal view of pharmacology. Annu Rev Pharmacol Toxicol. (1996) 36:1–33. 10.1146/annurev.pa.36.040196.000245 8725380

[64] Ribba B Boetsch C Nayak T Grimm HP Charo J Evers S. Prediction of the optimal dosing regimen using a mathematical model of tumor uptake for immunocytokine-based cancer immunotherapy. Clin Cancer Res. (2018) 24:3325–33. 10.1158/1078-0432.CCR-17-2953 29463551

[65] Lindauer A Valiathan C Mehta K Sriram V de Greef R Elassaiss-Schaap J. Translational pharmacokinetic/pharmacodynamic modeling of tumor growth inhibition supports dose-range selection of the anti-PD-1 antibody pembrolizumab: translational pharmacokinetic/pharmacodynamic modeling. CPT Pharmacomet Syst Pharmacol. (2017) 6:11–20. 10.1002/psp4.12130 27863176 PMC5270293

[66] Chen X Haddish-Berhane N Moore P Clark T Yang Y Li H. Mechanistic projection of first-in-human dose for bispecific immunomodulatory P-Cadherin LP-DART: an integrated PK/PD modeling approach. Clin Pharmacol Ther. (2016) 100:232–41. 10.1002/cpt.393 27170541

[67] Parra-Guillen ZP Berraondo P Grenier E Ribba B Troconiz IF. Mathematical model approach to describe tumour response in mice after vaccine administration and its applicability to immune-stimulatory cytokine-based strategies. AAPS J. (2013) 15:797–807. 10.1208/s12248-013-9483-5 23605806 PMC3691429

[68] Kosinsky Y Dovedi SJ Peskov K Voronova V Chu L Tomkinson H. Radiation and PD-(L)1 treatment combinations: immune response and dose optimization via a predictive systems model. J Immunother Cancer. (2018) 6:17. 10.1186/s40425-018-0327-9 29486799 PMC5830328

[69] Kosinsky Y Chu L Peskov K Voronova V Borodovsky A Woessner R Abstract 2098: quantitative modeling as a systematic approach for drug combination evaluation in immuno-oncology. (IO). Cancer Res. (2018) 78:2098 10.1158/1538-7445.AM2018-2098

[70] Serre R Benzekry S Padovani L Meille C Andre N Ciccolini J. Mathematical modeling of cancer immunotherapy and its synergy with radiotherapy. Cancer Res. (2016) 76:4931–40. 10.1158/0008-5472.CAN-15-3567 27302167

[71] Poleszczuk JT Luddy KA Prokopiou S Robertson-Tessi M Moros EG Fishman M. Abscopal benefits of localized radiotherapy depend on activated T-cell trafficking and distribution between metastatic lesions. Cancer Res. (2016) 76:1009–18. 10.1158/0008-5472.CAN-15-1423 26833128

[72] Lai X Friedman A. Combination therapy of cancer with cancer vaccine and immune checkpoint inhibitors: a mathematical model. PLoS ONE. (2017) 12:e0178479. 10.1371/journal.pone.0178479 28542574 PMC5444846

[73] Lai X Friedman A. Combination therapy for melanoma with BRAF/MEK inhibitor and immune checkpoint inhibitor: a mathematical model. BMC Syst Biol. (2017) 11:70. 10.1186/s12918-017-0446-9 28724377 PMC5517842

[74] Peng H Zhao W Tan H Ji Z Li J Li K. Prediction of treatment efficacy for prostate cancer using a mathematical model. Sci Rep. (2016) 6:21599. 10.1038/srep21599 26868634 PMC4751505

[75] Kronik N Kogan Y Vainstein V Agur Z. Improving alloreactive CTL immunotherapy for malignant gliomas using a simulation model of their interactive dynamics. Cancer Immunol Immunother. (2008) 57:425–39. 10.1007/s00262-007-0387-z 17823798 PMC11030586

[76] Cappuccio A Elishmereni M Agur Z. Cancer immunotherapy by interleukin-21: potential treatment strategies evaluated in a mathematical model. Cancer Res. (2006) 66:7293–300. 10.1158/0008-5472.CAN-06-0241 16849579

[77] Kronik N Kogan Y Elishmereni M Halevi-Tobias K Vuk-Pavlović S Agur Z. Predicting outcomes of prostate cancer immunotherapy by personalized mathematical models. PLoS ONE. (2010) 5:e15482. 10.1371/journal.pone.0015482 21151630 PMC2999571

[78] Kim R Woods T II Radunskaya A Mathematical modeling of tumor immune interactions: a closer look at the role of a PD-L1 inhibitor in cancer immunotherapy. SPORA J Biomath. (2018) 4:25–41. 10.30707/SPORA4.1Radunskaya

[79] Gong C Milberg O Wang B Vicini P Narwal R Roskos L. A computational multiscale agent-based model for simulating spatio-temporal tumour immune response to PD1 and PDL1 inhibition. J R Soc Interface. (2017) 14:20170320. 10.1098/rsif.2017.0320 28931635 PMC5636269

[80] Kather JN Poleszczuk J Suarez-Carmona M Krisam J Charoentong P Valous NA. *In silico* modeling of immunotherapy and stroma-targeting therapies in human colorectal cancer. Cancer Res. (2017) 77:6442–52. 10.1158/0008-5472.CAN-17-2006 28923860

[81] Kather JN Charoentong P Suarez-Carmona M Herpel E Klupp F Ulrich A. High-throughput screening of combinatorial immunotherapies with patient-specific *in silico* models of metastatic colorectal cancer. Cancer Res. (2018) 78:5155–63. 10.1158/0008-5472.CAN-18-1126 29967263

[82] Venkatakrishnan K Ecsedy J. Enhancing value of clinical pharmacodynamics in oncology drug development: an alliance between quantitative pharmacology and translational science. Clin Pharmacol Ther. (2017) 101:99–113. 10.1002/cpt.544 27804123

[83] Zhao X Wang X Feng Y Agrawal S Shah D Development of Antibody-Based Therapeutics: Application of PK-PD Modeling and Simulation Approaches for Immuno-Oncology Drugs. (2018). Available online at: 10.1007/978-981-13-0496-5 (accessed December 11, 2018).

[84] Stroh M Carlile D Li C-C Wagg J Ribba B Ramanujan S. Challenges and opportunities for quantitative clinical pharmacology in cancer immunotherapy: something old, something new, something borrowed, and something blue: something old, something new, something borrowed, and something blue. CPT Pharmacomet Syst Pharmacol. (2015) 4:495–7. 10.1002/psp4.12014 26451328 PMC4592528

[85] de Greef R Elassaiss-Schaap J Chatterjee M Turner D Ahamadi M Forman M Pembrolizumab: role of modeling and simulation in bringing a novel immunotherapy to patients with melanoma: modeling and simulation of Pembrolizumab. CPT Pharmacomet Syst Pharmacol. (2017) 6:5–7. 10.1002/psp4.12131 PMC5270292 27653180

[86] Nayak S Sander O Al-Huniti N de Alwis D Chain A Chenel M. Getting innovative therapies faster to patients at the right dose: impact of quantitative pharmacology towards first registration and expanding therapeutic use. Clin Pharmacol Ther. (2018) 103:378–83. 10.1002/cpt.978 29330855 PMC5838712

[87] Zhao X Suryawanshi S Hruska M Feng Y Wang X Shen J. Assessment of nivolumab benefit–risk profile of a 240-mg flat dose relative to a 3-mg/kg dosing regimen in patients with advanced tumors. Ann Oncol. (2017) 28:2002–8. 10.1093/annonc/mdx235 28520840 PMC5834087

[88] Freshwater T Kondic A Ahamadi M Li CH de Greef R de Alwis D. Evaluation of dosing strategy for pembrolizumab for oncology indications. J Immunother Cancer. (2017) 5:43. 10.1186/s40425-017-0242-5 28515943 PMC5433037

[89] Charych D Khalili S Dixit V Kirk P Chang T Langowski J. Modeling the receptor pharmacology, pharmacokinetics, and pharmacodynamics of NKTR-214, a kinetically-controlled interleukin-2. (IL2) receptor agonist for cancer immunotherapy. PLoS ONE. (2017) 12:e0179431. 10.1371/journal.pone.0179431 28678791 PMC5497954

[90] Zappasodi R Merghoub T Wolchok JD. Emerging concepts for immune checkpoint blockade-based combination therapies. Cancer Cell. (2018) 33:581–98. 10.1016/j.ccell.2018.03.005 29634946 PMC5896787

[91] Robertson-Tessi M El-Kareh A Goriely A. A mathematical model of tumor–immune interactions. J Theor Biol. (2012) 294:56–73. 10.1016/j.jtbi.2011.10.027 22051568

[92] Kirk PDW Babtie AC Stumpf MPH. Systems biology. (un)certainties. Science. (2015) 350:386–8. 10.1126/science.aac9505 26494748

[93] Davidian M Giltinan DM Nonlinear models for repeated measurement data: an overview and update. J Agric Biol Environ Stat. (2003) 8:387–419. 10.1198/1085711032697

[94] Kotecha R Kim JM Miller JA Chao ST Mohammadi AM Peereboom D Stereotactic radiosurgery (SRS) with immune checkpoint inhibitor therapy. (ICI) for patients with brain metastasis (BM): the impact of timing and sequencing. Int J Radiat Oncol. (2018) 102:e345–6. 10.1016/j.ijrobp.2018.07.1051

[95] Lehrer EJ Peterson JL Zaorsky NG Brown PD Sahgal A Chiang VL. Single versus multifraction stereotactic radiosurgery for large brain metastases: an international meta-analysis of 24 trials. Int J Radiat Oncol. (2018) 103:618–30. 10.1016/j.ijrobp.2018.10.038 30395902

[96] De Boer RJ Perelson AS Quantifying T lymphocyte turnover. J Theor Biol. (2013) 327:45–87. 10.1016/j.jtbi.2012.12.025 23313150 PMC3640348

[97] Tang C Welsh JW de Groot P Massarelli E Chang JY Hess KR. Ipilimumab with stereotactic ablative radiation therapy: phase I results and immunologic correlates from peripheral T cells. Clin Cancer Res. (2017) 23:1388–96. 10.1158/1078-0432.CCR-16-1432 27649551 PMC5355002

[98] Rothschilds AM Wittrup KD. What, why, where, and when: bringing timing to immuno-oncology. Trends Immunol. (2019) 40:12–21. 10.1016/j.it.2018.11.003 30545676

[99] Day D Siu LL. Approaches to modernize the combination drug development paradigm. Genome Med. (2016) 8:115. 10.1186/s13073-016-0369-x 27793177 PMC5084460

[100] Bui NQ Kummar S. Evolution of early phase clinical trials in oncology. J Mol Med. (2018) 96:31–8. 10.1007/s00109-017-1612-7 29177698

[101] Woodcock J LaVange LM. Master protocols to study multiple therapies, multiple diseases, or both. N Engl J Med. (2017) 377:62–70. 10.1056/NEJMra1510062 28679092

[102] Simonsen KL Fracasso PM Bernstein SH Wind-Rotolo M Gupta M Comprelli A. The fast real-time assessment of combination therapies in immuno-oncology (FRACTION) program: innovative, high-throughput clinical screening of immunotherapies. Eur J Cancer. (2018) 103:259–66. 10.1016/j.ejca.2018.07.127 30292142

[103] Wages NA Chiuzan C Panageas KS. Design considerations for early-phase clinical trials of immune-oncology agents. J Immunother Cancer. (2018) 6:81. 10.1186/s40425-018-0389-8 30134959 PMC6103998

[104] Gibney GT Weiner LM Atkins MB. Predictive biomarkers for checkpoint inhibitor-based immunotherapy. Lancet Oncol. (2016) 17:e542–51. 10.1016/S1470-2045(16)30406-5 27924752 PMC5702534

[105] Wargo JA Reddy SM Reuben A Sharma P. Monitoring immune responses in the tumor microenvironment. Curr Opin Immunol. (2016) 41:23–31. 10.1016/j.coi.2016.05.006 27240055 PMC5257261

[106] Galon J Bruni D. Approaches to treat immune hot, altered and cold tumours with combination immunotherapies. Nat Rev Drug Discov. (2019) 18:197–218. 10.1038/s41573-018-0007-y 30610226

[107] Pagès F Mlecnik B Marliot F Bindea G Ou F-S Bifulco C. International validation of the consensus Immunoscore for the classification of colon cancer: a prognostic and accuracy study. Lancet. (2018) 391:2128–39. 10.1016/S0140-6736(18)30789-X 29754777

[108] Finotello F Eduati F. Multi-omics profiling of the tumor microenvironment: paving the way to precision immuno-oncology. Front Oncol. (2018) 8:430. 10.3389/fonc.2018.00430 30345255 PMC6182075

[109] Galon J Angell HK Bedognetti D Marincola FM. The continuum of cancer immunosurveillance: prognostic, predictive, and mechanistic signatures. Immunity. (2013) 39:11–26. 10.1016/j.immuni.2013.07.008 23890060

[110] Paré L Pascual T Seguí E Teixidó C Gonzalez-Cao M Galván P Rodríguez A. Association between PD1 mRNA and response to anti-PD1 monotherapy across multiple cancer types. Ann Oncol. (2018) 29:2121–8. 10.1093/annonc/mdy335 30165419

[111] Ayers M Lunceford J Nebozhyn M Murphy E Loboda A Kaufman DR Albright A. IFN-γ-related mRNA profile predicts clinical response to PD-1 blockade. J Clin Invest. (2017) 127:2930–40. 10.1172/JCI91190 28650338 PMC5531419

[112] Li B Cui Y Diehn M Li R. Development and validation of an individualized immune prognostic signature in early-stage nonsquamous non–small cell lung cancer. JAMA Oncol. (2017) 3:1529–37. 10.1001/jamaoncol.2017.1609 28687838 PMC5710196

[113] Morrison C Pabla S Conroy JM Nesline MK Glenn ST Dressman D. Predicting response to checkpoint inhibitors in melanoma beyond PD-L1 and mutational burden. J Immunother Cancer. (2018) 6:32. 10.1186/s40425-018-0344-8 29743104 PMC5944039

[114] Duruisseaux M Martínez-Cardús A Calleja-Cervantes ME Moran S Castro de Moura M Davalos V. Epigenetic prediction of response to anti-PD-1 treatment in non-small-cell lung cancer: a multicentre, retrospective analysis. Lancet Respir Med. (2018) 6:771–81. 10.1016/S2213-2600(18)30284-4 30100403

[115] Auslander N Zhang G Lee JS Frederick DT Miao B Moll T Robust prediction of response to immune checkpoint blockade therapy in metastatic melanoma. Nat Med. (2018) 24:1545–9. 10.1038/s41591-018-0157-9 30127394 PMC6693632

[116] Jiang P Gu S Pan D Fu J Sahu A Hu X. Signatures of T cell dysfunction and exclusion predict cancer immunotherapy response. Nat Med. (2018) 24:1550–8. 10.1038/s41591-018-0136-1 30127393 PMC6487502

[117] Giladi A Amit I. Single-cell genomics: a stepping stone for future immunology discoveries. Cell. (2018) 172:14–21. 10.1016/j.cell.2017.11.011 29328909

[118] Papalexi E Satija R. Single-cell RNA sequencing to explore immune cell heterogeneity. Nat Rev Immunol. (2017) 18:35–45. 10.1038/nri.2017.76 28787399

[119] Tirosh I Izar B Prakadan SM Wadsworth MH Treacy D Trombetta JJ. Dissecting the multicellular ecosystem of metastatic melanoma by single-cell RNA-seq. Science. (2016) 352:189–96. 10.1126/science.aad0501 27124452 PMC4944528

[120] Lavin Y Kobayashi S Leader A Amir ED Elefant N Bigenwald C. Innate immune landscape in early lung adenocarcinoma by paired single-cell analyses. Cell. (2017) 169:750–65. 10.1016/j.cell.2017.04.014 28475900 PMC5737939

[121] Zheng C Zheng L Yoo J-K Guo H Zhang Y Guo X. Landscape of infiltrating T cells in liver cancer revealed by single-cell sequencing. Cell. (2017) 169:1342–56. 10.1016/j.cell.2017.05.035 28622514

[122] Jerby-Arnon L Shah P Cuoco MS Rodman C Su M-J Melms JC. A cancer cell program promotes T cell exclusion and resistance to checkpoint blockade. Cell. (2018) 175:984–97. 10.1016/j.cell.2018.09.006 30388455 PMC6410377

[123] Sade-Feldman M Yizhak K Bjorgaard SL Ray JP de Boer CG Jenkins RW. Defining T cell states associated with response to checkpoint immunotherapy in melanoma. Cell. (2018) 175:998–1013. 10.1016/j.cell.2018.10.038 30388456 PMC6641984

[124] Lesterhuis WJ Bosco A Millward MJ Small M Nowak AK Lake RA. Dynamic versus static biomarkers in cancer immune checkpoint blockade: unravelling complexity. Nat Rev Drug Discov. (2017) 16:264–72. 10.1038/nrd.2016.233 28057932

[125] Łuksza M Riaz N Makarov V Balachandran VP Hellmann MD Solovyov A. A neoantigen fitness model predicts tumour response to checkpoint blockade immunotherapy. Nature. (2017) 551:517–20. 10.1038/nature24473 29132144 PMC6137806

[126] Balachandran VP Łuksza M Zhao JN Makarov V Moral JA Remark R. Identification of unique neoantigen qualities in long-term survivors of pancreatic cancer. Nature. (2017) 551:512–6. 10.1038/nature24462 29132146 PMC6145146

[127] Roh W Chen P-L Reuben A Spencer CN Prieto PA Miller JP. Integrated molecular analysis of tumor biopsies on sequential CTLA-4 and PD-1 blockade reveals markers of response and resistance. Sci Transl Med. (2017) 9:eaah3560. 10.1126/scitranslmed.aah3560 28251903 PMC5819607

[128] Havel JJ Chowell D Chan TA. The evolving landscape of biomarkers for checkpoint inhibitor immunotherapy. Nat Rev Cancer. (2019) 19:133–50. 10.1038/s41568-019-0116-x 30755690 PMC6705396

[129] Chiacchio F Pennisi M Russo G Motta S Pappalardo F. Agent-based modeling of the immune system: netlogo, a promising framework. BioMed Res Int. (2014) 2014:1–6. 10.1155/2014/907171 24864263 PMC4016927

[130] Stéphanou A Volpert V Hybrid modelling in biology: a classification review. Math Model Nat Phenom. (2016) 11:37–48. 10.1051/mmnp/201611103

[131] Pappalardo F Lollini P-L Castiglione F Motta S. Modeling and simulation of cancer immunoprevention vaccine. Bioinformatics. (2005) 21:2891–7. 10.1093/bioinformatics/bti426 15817697

[132] Pappalardo F Motta S Lollini P-L Mastriani E. Analysis of vaccine's schedules using models. Cell Immunol. (2006) 244:137–40. 10.1016/j.cellimm.2007.03.002 17442286

[133] Brenner S. Sequences and consequences. Philos Trans R Soc B Biol Sci. (2010) 365:207–12. 10.1098/rstb.2009.0221 20008397 PMC2842711

[134] Emens LA Butterfield LH Hodi FS Marincola FM Kaufman HL. Cancer immunotherapy trials: leading a paradigm shift in drug development. J Immunother Cancer. (2016) 4:42. 10.1186/s40425-016-0146-9 27437105 PMC4949883

